# Effect of Waste Basalt Fines and Recycled Concrete Components on Mechanical, Water Absorption, and Microstructure Characteristics of Concrete

**DOI:** 10.3390/ma15134385

**Published:** 2022-06-21

**Authors:** Ibrahim A. Sharaky, Ahmed S. Elamary, Yasir M. Alharthi

**Affiliations:** Civil Engineering Department, College of Engineering, Taif University, P.O. Box 11099, Taif 21944, Saudi Arabia; a.elamary@tu.edu.sa (A.S.E.); y.harthi@tu.edu.sa (Y.M.A.)

**Keywords:** fine recycled aggregate, concrete strengths, waste basalt fines, recycled concrete powder, water absorption

## Abstract

In this paper, the recycled fine aggregates and powders produced from crushing old basaltic concrete and natural basalt were used to produce new concrete. The sand was partially replaced by two types of recycled wastes at five percentages: 0%, 20%, 40%, 60%, and 80%. The cement was partially replaced by recycled powders and silica fume (SF) at four percentages: 0, 5%, 10%, and 20%. The concrete strengths and water absorption were obtained at several curing ages. The obtained results emphasized the positive effects of increasing the curing time on enhancing the concrete properties, regardless of the types or the waste sources. Moreover, the recycled powders retarded the hydration reaction. In addition, the recycled fine aggregates and powders could achieve about 99.5% and 99.3% of the ordinary concrete strength and enhance the tensile strength. Furthermore, the mix containing 40% of recycled fine concrete aggregate diffused the highest contents of both calcium and silicate, which led to enhancing the interfacial transition zone (ITZ) and concrete properties, compared to the other tested mixes. Finally, the water absorption of all tested concrete mixes decreased with an increase in the curing age, while the mixes integrating 10% and 20% of SF experienced the lowest values of water absorption.

## 1. Introduction

Construction industry growth has become significant worldwide and its environmental impacts have increased rapidly due to the high demand for extracted raw materials. Concrete aggregates (sand and gravel) and water are the main materials used around the world, although their renewal rates are less than their usage. In 2017, about 45 billion tons of natural aggregates were extracted, while in 2025, the estimated extracted amount is predicted to rise to 66 billion tons. Moreover, the cement industries consume aggregates at a rate of about 40 billion tons/year worldwide [1,2,3]. To reduce the consumed amount of natural aggregates, research into the reuse of construction waste materials and the management of their disposal can decrease their removal costs and environmental impact (EI) [4]. Several countries have shown commitment to increasing the replacement of natural aggregates with recycled construction wastes (in Europe, about 10.93% of the used aggregates were from other sources (recycled, artificial, or landfilled) [2,5]. Decreasing landfill space, extraction energy, reducing the environmental impact, and saving natural aggregate (NA) resources are the main benefits of using recycled aggregate (RA) [6]. Moreover, the replacement of OPC with silica fume (SF) and Fly ash (FA) is necessary to decrease the environmental problems caused by OPC production [7,8]. The use of RA concrete (RAC) was limited to road substructures or non-structural applications. due to its high Water absorption (WA), its high porosity, and its low strength, compared to NA concrete (NAC) [9,10,11,12,13,14]. In previous studies [9,10,12], the NAC strength was obtained when the recycled concrete aggregate (RCA) percentage was up to 30%, or by using RCA produced from high-strength concrete (HSC). The previous finding assured the effectiveness of the RCA source in improving the obtained RAC strengths.

Ameliorating the environmental impact (EI) of concrete production became essential some time ago. Cement production and natural aggregate-extracting processes are one of the main hazardous gas sources (i.e., CO_2_, CO, and NO_x_). Using equipment with low fuel combustion or alternative materials instead of cement and natural aggregates (i.e., fly ash and RCA) are two alternatives for reducing hazardous gas emissions. To reduce the EI of cement and aggregate production, fly ash was used as a replacement material (i.e., sintered fly ash instead of coarse aggregate, cenosphere fly ash instead of sand, and fly ash instead of cement) [15,16]. Additionally, the replacement of NA with RCA reduced both the EI and the cost [17,18,19]. For instance, the EI and cost were reduced by 8% and 9%, respectively, when the NA was replaced by 30% RCA, while at 100% RCA, the EI and cost were reduced by 23% and 28%, respectively [17,20]. In an earlier study [21], when RCA was used instead of NA, the EI and cost reductions reached 50.8% and 68.1%, respectively. Consequently, in earlier studies by [22,23], maximum EI and cost efficiencies were achieved at 50% RCA and 80% RCA, respectively.

Several researchers have previously studied the influence of recycled fine concrete aggregate (RFCA) on the mechanical behavior and durability of concrete [2,17,24,25,26,27,28,29]. In an earlier study [24], the compressive strength diminished by 15% and 30%, compared to NAC, when the natural sand replacement ratios were 25% RFCA and 100% RFCA, respectively; in contrast, the incorporation of RFCA into concrete increased its shrinkage. In [25,30], the reported results relating to the effects of RFCA on the concrete strength and shrinkage supported the obtained results reported in [24]. Conversely, in [18], when incorporating up to 30% RFCA, the obtained strength was nearly unchanged, compared to the strength of NAC. In contrast to the carburization resistance, the chloride penetration and WA of RAC increased as the RFCA replacement ratios increased [26]. Consequently, the incorporation of RFCA to the cement mortar grout had the same effect on its strengths (compressive and flexural) as in the case of RAC [28,29]. Moreover, the RAC-integrated RFCA was greatly affected by the means of its production [27]. Natural sand was also replaced by recycled glass sand (RGS), rubber (RA), individual plastics (PA), and glass (GA). Concrete integrating 20% of RGS had the same NAC strength after 7 days while raising the RGS percentage slightly decreased the concrete’s strength. Consequently, the tensile strength of concrete incorporating RGS was 8–11% of its compressive strength [31]. Conversely, the partial replacement of sand with PA and RA reduced the concrete strength. The compressive and tensile strengths for concrete integrating 30% PA were about 50% and 63% of the corresponding NAC strengths, respectively, while the concrete integrating 30% RA gained about 65% of the NAC strength [32].

Several supplementary cement-based materials (SCM), such as metakaolin, rice husk ash, waste glass, GGBFS, and silica fume have been implemented during the last decade as a cement replacement [33,34,35,36,37,38,39,40,41]. Although fly ash and GGBFS have been annually produced at a rate of one billion tons and 360 billion tons, respectively, there is a worldwide need to find other SCM materials to be used as cement replacements, to meet the rising demand [38]. The high reactivity of SF with calcium hydroxide, formed through cement hydration, allows it to act as a suitable replacement SCM for cement. Due to the latent hydraulic and pozzolanic properties of GGBFS, it has been largely used as a supplementary cement-based material (SCM) for producing cement and concrete. The properties of GGBFS also enhanced the immediate and long-term properties of hardened concrete [42]. Several researchers also studied the effect of using GGBFS on the properties of RCA [43,44,45]. The RAC that integrated 50% RCA and 30% GGBFS achieved nearly the same mechanical characteristics as NAC [43], while concrete cast with 50% RCA and 40% GGBFS resulted in concrete with good mechanical and physical properties [44]. Conversely, in the case of 60% GGBFS cement replacement, the RAC strength decreased because of the low hydraulic activity of GGBFS compared to cement. The hydrated lime increased the activity of GGBFS and enhanced the RAC properties [46,47,48].

In a previous study [49], the machine-made sand concrete (MSC) indicated an increase in its compressive strength at low content levels of aggregate micro fines (AMF); then, the strength decreased with a further increase in AMF content. Conversely, the AMF content evinced the opposite effect on the chloride and water permeability coefficients of the MSC. Moreover, the three dimension (3D)-printing mortar mixtures integrated with AMF and FA show lower and higher strengths than those without AMF, before 28 and after 60 days of curing, respectively [50]. Furthermore, the effect of AMF on the bulk density of mortar has also been studied [51,52]. The AMF enhanced the density and mechanical properties of fresh and hardened mortar, respectively [51]. The optimal AMF content depended on the water/cement (W/C) and sand/cement (S/C) ratios [52]. The seashell powder enhanced the rheological properties of combined cement mixes when it partially replaced cement [53,54], while the water-binder ratio of the mixes was reduced with the addition of the seashell powder [53]. Moreover, the addition of seashell powder improved the hydration capacity of cement and the strength of hardened cement mixes [54].

The basalt aggregates and powder improved the concrete’s properties, while silica fume (SF), basalt powder, and ordinary Portland cement (OPC) had nearly the same composition [55,56,57]. The combined use of SF and steel fibers increased the bending strength of RAC exposed to fire, while 4% SF was the optimal content to enhance the fracture properties of RAC with steel fibers [58]. The effect of natural basalt powder (PB) on the cement mortar and the concrete’s properties has previously been studied [59,60,61,62]. In one study [59], the cement was partially replaced by PB (the replacement ratio was 0 to 30%). The PB delayed the setting time and the progress of the hydration process of the paste. Consequently, the paste integrated with PB developed lower compressive strength than that with pure cement at an early age, while the strengths were closer at a later age. In another study [60], PB was used as the SCM (PB ≤ 0.5 wt %) to modify the porosity, permeability, chemical resistance, and mechanical characteristics of cement paste, while when the PB was more than 0.5 wt %, the opposite effects on the cement paste properties were obtained. Moreover, in [61], the effect of SF, slag, limestone, and PB on the cement paste properties was verified. At early ages, SF and slag had higher pozzolanic activity than PB, while the pozzolanic activity of PB increased afterward. Besides, PB was better than SF, limestone, and slag as it showed a good filling ability to enhance hydration and physico-mechanical properties of cement Conversely, in [62], the replacement of fine aggregates with PB enhanced the concrete’s characteristics. The concrete strengths (both compressive and flexural) increased when PB was incorporated into the mix. Moreover, PB acted as a micro-filler in the concrete, to produce a denser and stronger Interfacial Transition Zone (ITZ) than that of concrete without PB. Recycled aggregates (both fine and coarse) and powders produced from old basaltic concrete were used to replace natural concrete components in another study [63].

The above review reports the limited research that has studied the incorporation of recycled basaltic concrete (concrete cast with basalt aggregates). Moreover, the waste fines produced during the production of coarse basalt aggregate were usually used for leveling the roads. The incorporation of the previous fines was slightly used in concrete. In this study, the fines produced from the crushing of old basaltic concrete or those produced from the production stations of the basalt aggregates were used to replace the fine NA and cement. All the obtained results were compared, to discuss the effects of curing time and the sources of recycled fine aggregates and powders on the strengths, microstructure, and WA of concrete.

## 2. Research Importance

The fines from old basaltic concrete were sieved to form recycled fine concrete aggregate and fine basaltic concrete powder (RFCA and RCP), respectively. Subsequently, the waste fines produced from the production station of basalt aggregate were sieved to form recycled fine natural aggregate and aggregate micro-fines (RFNA and AMF), respectively. The RFCA and RFNA were used to substitute the natural sand at five percentages (0%, 20%, 40%, 60%, and 80%). Then, the cement was partially replaced by RCP, AMF, and SF at four percentages (0%, 5%, 10%, and 20%). Finally, the effects of RFCA, FRCP, RCP, AMF, and SF on the microstructure properties, mechanical properties, and water absorption of the resulting concretes were studied.

## 3. Experimental Work

### 3.1. Material Properties

#### 3.1.1. Aggregates

**Figure 1:**
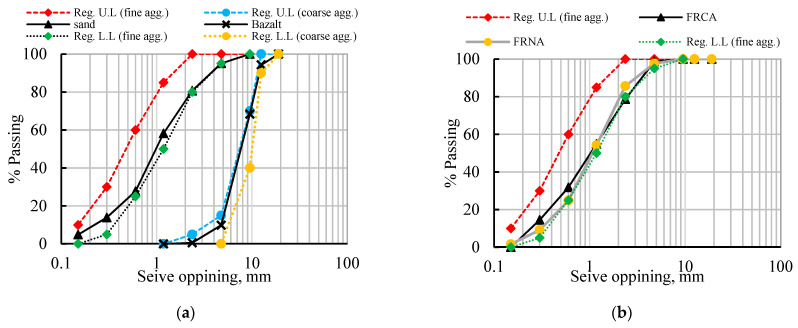
Sieve analysis for natural and recycled aggregates: (**a**) NA; (**b**) RFCA and RFNA.

Crushed basalt with a maximum nominal size (MNS) of 12.5 mm was used as a coarse aggregate. The basalt grading curve was adopted to comply with ASTM C33 regulations [64] (Figure 1a). The natural sand, RFCA, and RFNA comprised the fine aggregates. The fineness modulus (FM) of natural sand was experimentally obtained (FM = 3.0). The RFCA and RFNA were obtained by sieving the crushed recycled basaltic concrete and the wastes of the crushing station of basalt stones (the means by which basalt aggregate is produced), respectively. The RFCA and RFNA fines passed through sieve No. 4 (sieve openings = 4.75 mm microns) and remained on sieve No. 100 (sieve openings = 150 microns). Consequently, the grading of RFCA and RFNA were assumed to have the same FM as sand and to comply with the ASTM C33 regulations [64] (Figure 1a,b). The physical properties of crushed basalt, sand, RFCA, and RFNA are listed in Table 1.

#### 3.1.2. Cement and Pozzolanic Materials

**Figure 2:**
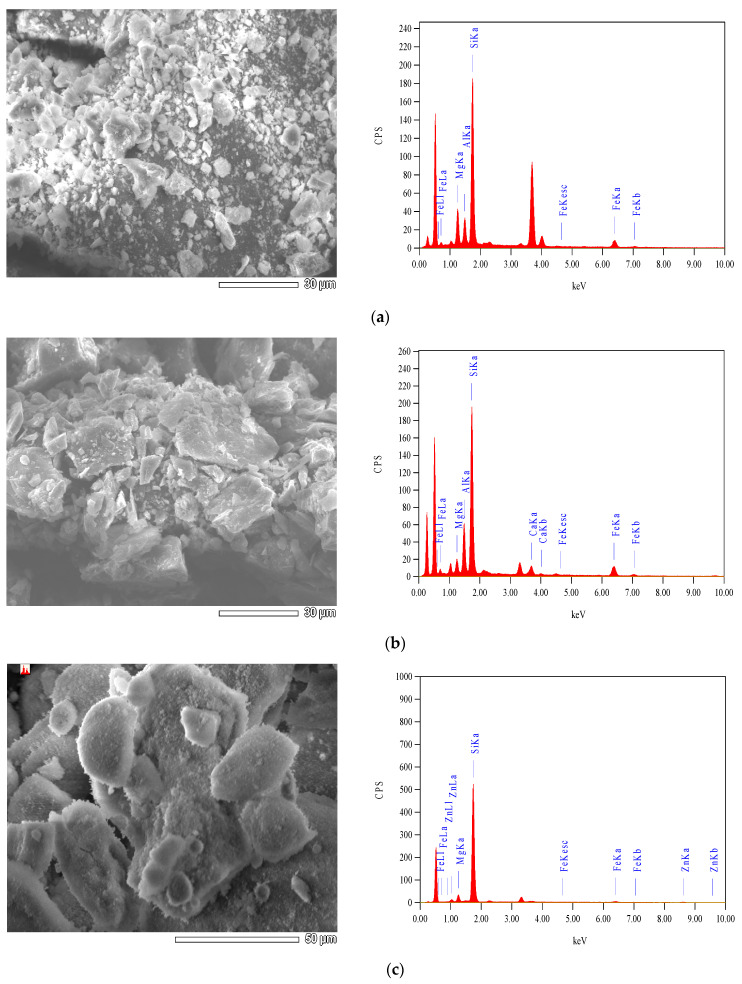
The EDS of the recycled concrete powder, recycled natural powder, and silica fume: (**a**) RCP; (**b**) AMF; (**c**) SF.

All prepared concrete mixes were cast using OPC. The RCP, AMF, and SF products were used to partially replace the OPC. The chemical composition of RCP, AMF, and SF were obtained using scanning electron microscopy (SEM), along with energy dispersive spectroscopy (EDS). The EDS analysis of the RCP, AMF, and SF are shown in Figure 2. Consequently, the chemical components of the OPC, RCP, AMF, and SF are listed in Table 2. The sum of SiO_2_, Fe_2_O_3_, and Al_2_O_3_ should be greater than 70% for the pozzolanic materials (ASTM C618). For RCP and AMF, the sum of the three components (SiO_2_, Fe_2_O_3_, and Al_2_O_3_) was 87.68% and 90.59%, respectively, which met the requirements for natural pozzolana (ASTM C618). Moreover, the main chemical component of RCP and AMF was silica (SiO_2_). The SF as a well-known industrial pozzolanic material (the sum of SiO_2_, Fe_2_O_3_, and Al_2_O_3_ were 93.2%; see Table 2), was used to replace the OPC; and the obtained results will be compared with those of RCP and AMF. 

### 3.2. Mix Design, Specimens, and Testing Methods

**Figure 3:**
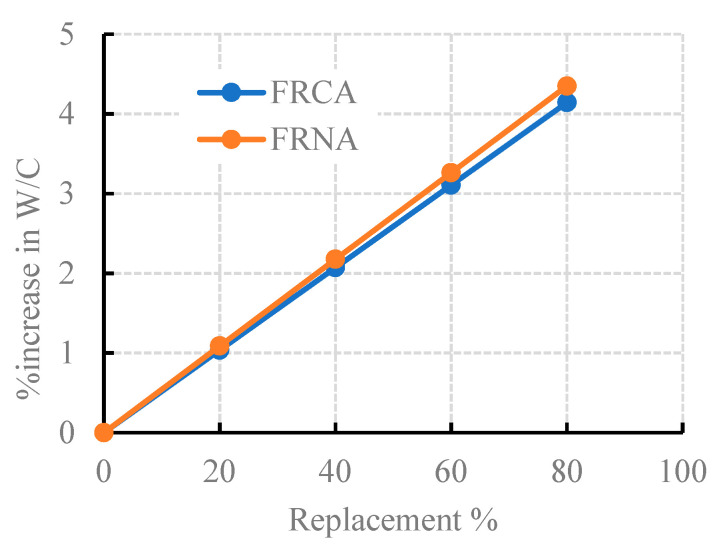
The percentage increase in the W/C ratio for RFCA and RFNA.

In this paper, the control mix (M0), cast with NA and cement, was designated according to the model in [65]. The target compressive strength at 28 days (*f_cu_*_,28_) was 35 MPa; the slump was 50–80 mm, while the W/C was 0.5. To study the source effect of recycled fine aggregates (RFCA and RFNA) and powders (RCP and AMF) on the concrete strength and WA, all other components of the concrete mix were kept constant. Although most of the previous researchers [18,51,53,65] neglected the WA and humidity factors of NA and RCA when designing concrete mixes, herein, only one mix (M0) was designed to neglect the WA and humidity of NA; the measured slump was in the designed range. Conversely, in the RAC mixes, the differences in the WA and humidity between sand, RFCA, and RFNA were considered. To obtain the same consistency of M0 for all mixes, extra water was added to the RAC mixes, as shown in Figure 3. As the recycled RFNA contained fines from weak and permeable stones, it absorbed more water than the RFCA. To produce sustainable RAC, seventeen mixes (M1 to M17) were prepared. For mixes M1–M4 and M8–M11, sand was partially replaced by RFCA and RFNA, respectively, at five percentages (0%, 20%, 40%, 60%, and 80%, respectively; see Table 3). Consequently, for mixes M5–M7, M12–M14, and M15–M17, cement was partly substituted by RCP, AMF, and SF, respectively, at four percentages (0%, 5%, 10%, and 20%, respectively; see Table 3).

**Figure 4:**
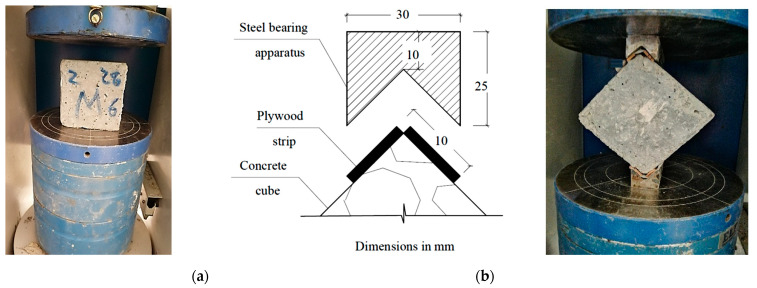
Details of the compression and diagonal cube-splitting tests: (**a**) compression test; (**b**) diagonal splitting test.

The authors tried to keep all factors constant except the replacement material, so no water reducers were added when the cement or NA was replaced by recycled materials or SF. The aggregates (NA, RFCA, and RFNA) were dry-mixed using a mechanical concrete mixer for 1 min. Subsequently, cement and other pozzolanic materials (if any) were added to the mixer; then, all components were dry-mixed for an additional 1 min. Afterward, the calculated water amount was steadily added to the mixer and a further 2 min of mixing was conducted. Twenty-one 100 × 100 × 100 mm^3^ cubes were cast from each mix, de-molded after one day, and submerged in water until testing. Three cubes were directly tested in compression (Figure 4a) at each curing age (7, 28, and 56 days), as is consistent with BS EN 12390-3 [66], and the results were averaged to obtain compressive strengths at 7 days (*f_cu_*_,7_), 28 days (*f_cu_*_,28_), and 56 days (*f_cu_*_,56_). The other three cubes were diagonally tested in compression (Figure 4b) at 28 days and 56 days to obtain the concrete tensile strengths *f_tu_*_,28_ and *f_tu_*_,56_, respectively, using Equation (1) [67]:

(1) $$\sigma_{max}=\frac{2P}{\pi bd}\left[{\left(1-\beta^{2}\right)}^{\frac{5}{3}}-0.0115\right]$$

where *b* and *d* are the cube width and diagonal length, respectively, while *P* is the failure load and *β* = 0.15. The cubes were loaded using a concrete-testing machine (capacity = 2000 tons) with loading rates of 4 and 3 kN/s for the compression and diagonal tests, respectively.

There are a limited number of standard methods used to measure the WA of concrete in laboratory situations. The most rigorous standard approaches are ASTM C1585 and ASTM C642 [68]. Moreover, BS 1881 [69] is used to evaluate the WA of concrete. The most simple and rapid tests used to measure the WA of concrete are those based on absorption. ASTM C642 [64] establishes the WA of concrete using two saturation methods, with no shape limitation for the specimens except the sample volume (≥350 cm^3^, approx = 800 g). In this study, the specimens were dried at 100–110 °C (time ≥ 24 h) then the oven-dry mass (A) was measured. Afterward, the specimens were immersed in water (time ≥ 28 h and temperature ≈ 21 °C), then the surface-dried samples’ weights (B) were measured (all weights were taken in grams). The WA was calculated for all mixes at 28 and 56 days, using Equation (2) [68]:

(2) $${\mathrm{WA}\;\%\;}_{\left(\mathrm{after}\;\mathrm{immersion}\;\mathrm{only}\right)}=\left[\frac{\left(B-A\right)}{\;A}\right]\times 100.$$

## 4. Results and Discussions

### 4.1. Compressive Strength

The measured compressive strengths at 7, 28, and 56 days are classified in Table 4. Consequently, the reduction in the compressive strength of the tested RAC mixes at 7 days (*µ*_7_), 28 days (*µ*_7_), and 56 days (*µ*_56_) is also summarized in Table 4. Moreover, the values for *µ*_28/7_% = *f_cu_*_,28_/*f_cu_*_,7_ × 100 and *µ*_56/28_% = *f_cu_*_,56_/*f_cu_*_,28_ × 100 for all mixes are also listed in Table 4. At each testing age, the percentage decrease in compressive strength of the tested RAC mixes compared to M0 (*µ_cu,age_*%) was also measured (see Equation (3)).

(3) $$\mu_{cu,age}\%=\frac{f_{cu,age}\left(\mathrm{RAC}\right)-f_{cu,age}\left(M0\right)}{f_{cu,age}\left(M0\right)}\times 100$$

Here, *f*_*cu*,*age*_ (RAC) and *f*_*cu*,*age*_ (M0) are the *f_cu_* values for the mix containing recycled materials and the control mix, respectively.

**Figure 5:**
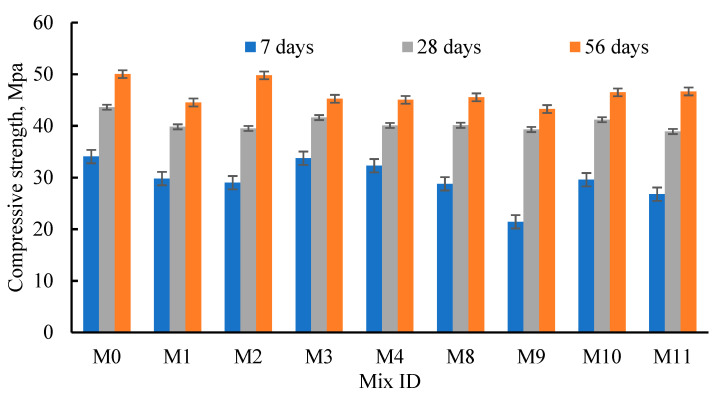
Effect of the RFCA and RFNA on concrete compressive strength.

The influence of fine recycled materials on concrete strength was studied (Figure 5). As the *T_c_* increased, the RAC strength improved for all mixes (Figure 5). Moreover, the *µ*_28/7_ values of the tested cubes were higher than the *µ*_56/28_ values (Table 4). This emphasized the higher hydration rate of cementitious materials (cement and the basalt powders) during the first 28 days compared to in later days. Consequently, the compressive strength at 28 days for M1 to M4 was higher than the strengths at 7 days. by about 23.33–36.14%, depending on the RFCA percentage. Conversely, for the same previous mixes (M1 to M4), as the Tc increased from 28 to 56 days, the strength enhanced by 8.8–26%, depending on the RFCA percentage. Moreover, regardless of the curing age, the concrete strength was greatly affected by the RFCA percentage (the strength generally decreased with sand replacement). At *T_c_* = 7 days, as the RFCA replaced sand by 20–80%, the strength was reduced by 1.0–14.8% (*µ*= 8.38% and *σ_µ_* = ±5.5%), compared to that of M0. Moreover, at Tc = 28 days, the concrete strength was reduced by 4.6–9.45% (*µ* = 7.68% and *σ_µ_* = ±1.58%), compared to that of M0, when the RFCA replaced sand by 20–80%. Furthermore, at Tc = 56 days, the strength reduction was about 0.50–11.0% (*µ* = 5.75% and *σ_µ_* = ±4.22%), compared to that of M0, when the RFCA replaced sand by 20–80%. The lowest reduction in strength values was obtained for M7, when tested at 7 and 28 days (*µ*_7_ = 1.0% and *µ*_28_ = 4.6%), and for M6, when tested at 56 days (*µ*_56_ = 0.50%). These lowest strength reductions support the use of RFCA as a good replacement for natural sand.

To discuss the effect of the fine RCA resources on the concrete strength, the RFNA was used to replace sand (M8–M11) by the same percentages as the RFCA (0–80%, Figure 5). The effect of *T_c_* on the strength of concrete integrating RFNA was nearly the same as that integrating RFCA (Table 3, Figure 5). At *T_c_* = 7 days, the strength reduction (*µ*_7_) for mixes integrating RFNA was 13.1–37.1%. Conversely, the *µ*_28_ for M8-M11 mixes was 5.5–10.8%, while the *µ*_56_ for M8-M11 mixes was 6.7–13.5%. The two mixes (M10 and M11) integrating high RFNA percentages experienced the lowest values of *µ*_56_ (7.1% and 6.7%, respectively). The mixes integrating RFCA showed better compressive strength results than those integrating RFNA (Figure 5). The high Sio2 contents in the RCA increased the bond between the concrete components. Sio2 reacted with the CH of cement and produced more calcium silicate hydrate (C-S-H) gel. These results support the use of high replacement ratios of RFNA and RFCA to replace the natural sand. After 28 days of curing, integrating the RFCA and RFNA instead of sand, the samples showed nearly the same *µ*_28_ percentage values.

**Figure 6:**
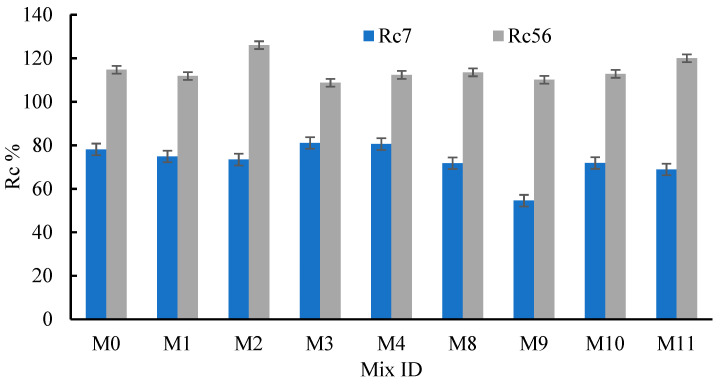
Effect of the RFNA and RFNA on Rc7 and Rc56.

Figure 6 shows a comparison between the strength gained at 7 and 56 days, compared to that at 28 days (Rc7% and Rc56%). The Rc7 values were 73.5–81.1% (*µ* = 77.6% and *σ_µ_* = ±3.03%) when the RFCA replaced sand by 0–80%. Consequently, the Rc56 values were 108.8–126.0% (*µ* = 114.7% and *σ_µ_* = ±5.95%) when RFCA replaced sand by 0–80%. The highest values of Rc7 and Rc56 were obtained for M3 and M2, respectively (Figure 6). Comparing the obtained results with those reported in [32] demonstrated the greater efficiency of RFCA in restoring the NAC’s strength than both RA and PA. In [70,71], a strength reduction of about 26–32% was reported when the RCA replaced NA. Moreover, the RAC showed a higher pore structure than NAC [72,73]. To compare the effect of the RFNA percentage on the gained strength values at 7 and 56 days, compared to that at 28 days, the Rc7 and Rc56 values were calculated (see Figure 6). From the figure, it can be seen that the Rc7 values were 54.6–78.1% (*µ* = 66.8% and *σ_µ_* = ±7.15%) when the RFNA replaced sand by 0–80%. In addition, the Rc56 percentages were 110.1–120.0% (*µ* = 114.24% and *σ_µ_* = ±3.24%) when the RFNA replaced sand by 0.0–80%. Comparing Rc7 and Rc56, in mixes integrating RFNA (except M9), with that of mix M0 emphasized the low effect of the RFNA percentage on the Rc7 and Rc56 values. From all the above tests, owing to the concrete strength results, basaltic RFCA and RFNA could be considered good replacement materials for the fine NA used to produce sustainable concrete, integrating high RFCA and RFNA contents and reducing the environmental impact and concrete production costs.

**Figure 7:**
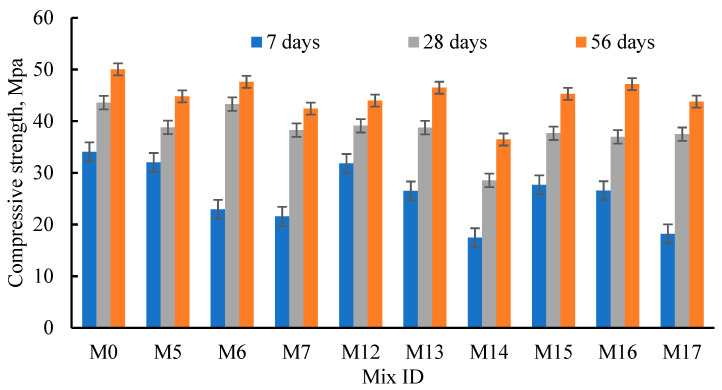
Effect of the RCP, AMF, and SF contents on concrete compressive strength.

To produce green concrete, RCP, AMF, and SF were used to partially replace the OPC. The OPC was partially substituted by RCP, AMF, and SF at four percentages (0%, 5%, 10%, and 20%). From Table 4 and Figure 7, the compressive strength was enhanced as Tc levels increased at all the replacement ratios. The effect of the replacement materials on the strength was greatly influenced by both the curing time and the replacement ratio. At 7 days, the concrete strength was reduced by 6.0–36.7%, 6.6–58.5%, and by 18.8–46.6% when the OPC was partially replaced by RCA, AMF, and SF, respectively. Conversely, at 28 days, the concrete strength diminished by 0.7–12.2%, 10.3–34.3%, and 13.6–15.2% when the OPC was partially replaced by RCA, AMF, and SF, respectively. Moreover, the attained values of *µ*_56_ were 4.8–15.2%, 7.1–27.1%, and 5.7–12.5% when the OPC was partially replaced by RCA, AMF, and SF, respectively. The low values of *µ*_28_ and *µ*_56_ compared to *µ*_7_ ensured the late hydration of RCP, AMF, and SF. Moreover, mixes M6 (10% RCP), M16 (5% AMF), and M19 (5% SF) showed the lowest values of *µ*_28_ (0.7%, 10.3% and 13.6%, respectively). Furthermore, mixes M6 (10% RCP), M17 (10% AMF) and M20 (10% SF) showed the lowest values of *µ*_56_ (4.8, 7.1, and 5.7%, respectively). At 28 days, the mixes integrating RCP, AMF, and SF exhibited about 99.3% (M6), 89.7% (M16), and 86.4% (M19) of that of M0, respectively. Consequently, at 56 days, the mixes integrating RCP, AMF, and SF exhibited about 95.2% (M6), 92.9% (M17), and 94.3% (M20) of that of M0, respectively. The previous findings demonstrated the pozzolanic effects of RCP, AMF, and SF.

**Figure 8:**
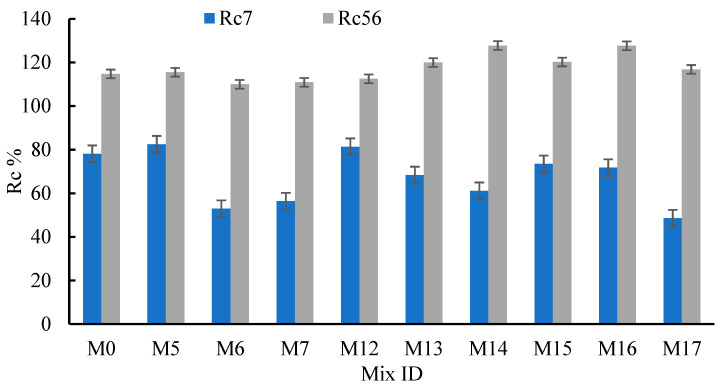
Effects of the RCP, AMF, and SF percentages on Rc7 and Rc56.

**Figure 9:**
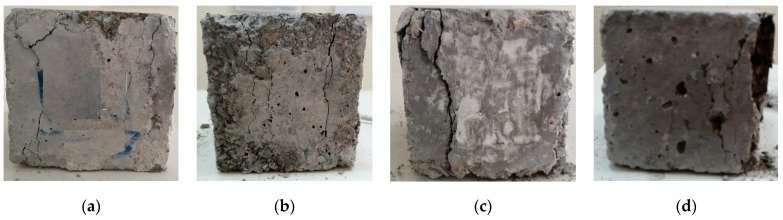
Compressive failure for tested cubes at 7, 28, and 56 days: (**a**) M7 at 7 days; (**b**) M9 at 7 days; (**c**) M9 at 28 days; (**d**) M9 at 56 days.

The effects of RCP, AMF, and SF on the achieved strength at 7 and 56 days, relative to that at 28 days, were calculated (Rc7 and Rc56, Figure 8). From the figure, it can be seen that Rc7 values were about 53.0–82.5%, 61.1–81.4%, and 48.6–73.5% for concrete integrating RCP, AMF, and SF, respectively. Consequently, the Rc56 values were 110–115.5%, 112.5–127.7%, and 116.8–127.6% for concrete integrating RCP, AMF, and SF, respectively. Comparisons between the SF, RCP, and AMF effects on the concrete strength supported the suitability of RCP and AMF as good replacement materials for OPC as they exhibited the same pozzolanic effects as the mineral material (SF). Moreover, the use of RCP and AMF to partially replace the OPC decreased their environmental impacts and reduced the concrete’s cost. The replacement of OPC at 5–25%, besides the addition of the recommended superplasticizers, enhanced the concrete’s strength [71,74,75]. The reported and obtained results support the use of superplasticizers with RCP and AMF as they may regulate their hydration and increase the concrete strength. The failures of the cubes tested in compression are shown in Figure 9.

### 4.2. Tensile Strength

**Figure 10:**
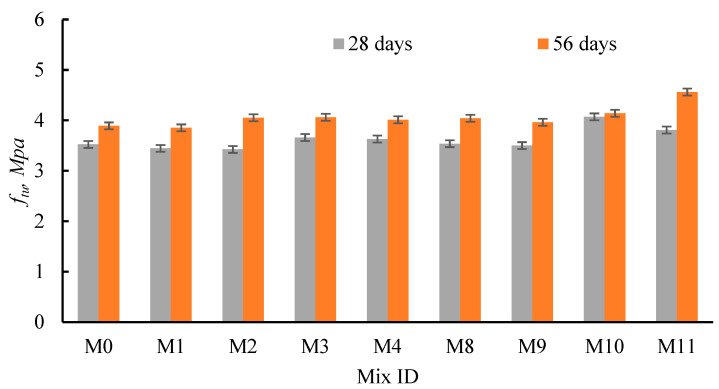
Effects of the RFCA and RFNA on concrete diagonal strength.

**Figure 11:**
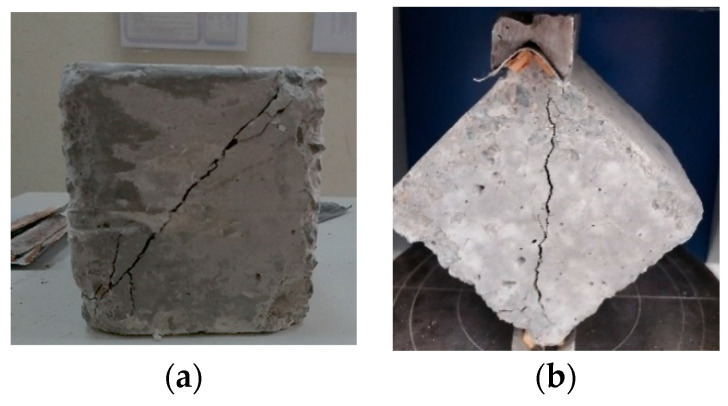
Tensile diagonal failure for certain cubes at 28 and 56 days: (**a**) M1 at 56 days; (**b**) M2 at 28 days.

The cubic diagonal test was used to evaluate the concrete tensile strength (*f_tu_*) for all concrete mixes. The tensile strengths at 28 and 56 days of curing (*f_tu_*_,28_ and *f_tu_*_,56_, respectively) are reported in Table 5 and Figure 10 and Figure 11. Consequently, the change in tensile strength with curing time (at 28 days = *µ_t_*_,28_, and at 56 days = *µ_t_*_,56_) due to the partial substitution of sand and cement with recycled materials is also presented in Table 5. Moreover, the effect of increasing the curing time from 28 to 56 days (*µ_t_*_56/28_) on the diagonal concrete strength for all mixes was also calculated (Table 5). Furthermore, the ratios between the tensile and compressive strengths due to the incorporation of the recycled materials at 28 days (*f_tu_*_,28_/*f_cu_*_,28_) and at 56 days (*f_tu_*_,56_/*f_cu_*_,56_%) were also evaluated (Table 5). The increase/decrease in *f_tu_* (*µ*_*t*,*date*_%) at each curing time was determined using Equation (4):

(4) $$\mu_{t,age}=\frac{f_{tu,age}\left(\mathrm{RAC}\right)-f_{tu,age}\left(M0\right)}{f_{tu,age}\left(M0\right)}\times 100$$

Here, *f*_*tu*,*age*_(RAC) and *f*_*tu*,*age*_(M0) are the *f_tu_* values for the mixes containing recycled materials and the control mix, respectively. The RFCA and RFNA replaced the natural sand at five percentages (0%, 20%, 40%, 60%, and 80%). The cubic diagonal strength depended on the type and percentage of the recycled materials, as well as curing time (Figure 10). As Tc increased from 28 to 56 days, the *f_tu_* values increased, regardless of the type and percentage of the recycled materials (Figure 10 and Table 5). Consequently, M6 and M12 experienced the highest *µ_t_*_56/28_ percentage values (19.80% and 14.3%, respectively). Moreover, at the same curing age, the mixes cast with RFCA and RFNA had higher diagonal strengths than M0, except for M5, M6, and M13 at 28 days (Figure 10). The values of *µ_t_*_28_ were −2.88–3.87%, while the *µ_t_*_56_ values were 0.26–5.40% for mixes integrating 20–80% RFCA. Moreover, the values of *µ_t_*_28_ were −0.68–15.50%, while the *µ*_*t*56_ values were 1.80–17.23% for mixes cast with 20–80% RFNA (Table 5). The addition of RFNA to the mixes had a greater effect than RFCA on enhancing the diagonal strength. The availability of the RCP or AMF attached to the RFCA and RFNA, respectively, besides the voids existing in the RFCA may be the reasons for this finding. Conversely, basaltic RFCA and RFNA might act as microfibers, bridging the internal macro cracks and increasing the tensile strength. When *f_tu_*_,28_/*f_cu_*_,28_% and *f_tu_*_,56_/*f_cu_*_,56_% for the mixes cast with RFCA and RFNA, compared with that of M0, the substantial effects of mixes integrating RFCA and RFNA on the tensile strength were greater than on the compressive strength. The pozzolanic effects of RCP and AMF might enhance the bonds between the concrete particles. As was reported in [72], cast concrete with RCA had a more highly porous structure than that cast with NA. 

Comparing the effects of both RFCA and RFNA on the concrete diagonal strength with that integrating RA and PA [32] supports the use of RFCA and RFNA to replace sand. The cubic diagonal failure of the tested cubes (Figure 11) followed the same shapes as were reported in [67].

**Figure 12:**
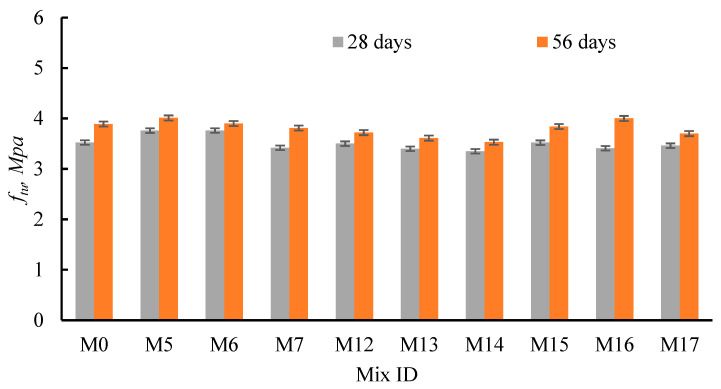
Effect of the RCP, AMF, and SF on concrete tensile strength.

To study the effect of RCP and AMF, compared with SF as a well-known pozzolanic material, on the tensile strength, cubes cast with RCP, AMF, and SF were tested (Figure 12 and Table 5). For all the tested mixes, the diagonal strength increased as the TC increased, regardless of the recycled powder type and content. As the curing time was raised from 28 to 56 days, the tensile strength was enhanced by 3.7–11.4%, 5.4–6.3%, and 7.0–17% for mixes integrating RCP, AMF, and SF, respectively. This ensured the difference in the rates of hydration of RCA, RCP, and SF over time and the remaining amount of un-hydrated cement. Moreover, increasing the substitution ratios of RCP, AMF, and SF decreased the diagonal strength. Among the three tested powders, mixes integrating RCP increased the diagonal strength up to RCP = 10% at 28 days. Consequently, the diagonal strength at 28 days increased by 6.81% and 6.81%, while at 56 days, it increased by 3.09% and 0.26%, compared to M0, when RCP replaced cement by 5% and 10%, respectively. Conversely, the diagonal strength at 28 days decreased by −0.56% and −3.5%, while at 56 days, it decreased by −4.37% and −7.17%, compared to M0, when AMF replaced cement by 5% and 10%, respectively. Moreover, the diagonal strength at 28 days decreased by 0.0% and −3.3%, compared to M0, when SF replaced cement by 5% and 10%, respectively, while at 56 days, it decreased by −1.3% and increased by 2.9%, compared to M0, when SF replaced cement by 5% and 10%, respectively. On the other hand, when RCP, AMF, and SF replaced cement by 20%, the diagonal strength decreased by −2.92%, 4.80%, and −1.90% at 28 days, while at 56 days, it decreased by −2.05%, −9.25% and −5.00% compared to M0, respectively. The previous observations revealed the substantial effect of cement powder content in the RCP on the tensile strength.

The values of *f_tu_*_,28_/*f_cu_*_,28_ and *f_tu_*_,56_/*f_cu_*_,56_ percentages ensured a nearly constant effect of SF on both compressive and tensile strengths for all SF percentages. In contrast, the values of *f_tu_*_,28_/*f_cu_*_,28_ and *f_tu_*_,56_/*f_cu_*_,56_ percentages depended on the content of RCP and AMF in the mix (these observations are supported by those given in [76]). Adding SF by 8–12% as a cement replacement augmented the tensile strength [77]; similar effects of SF (0–25%) on the tensile strength were also reported [74]. In contrast, substituting SF at 5 and 10% of the cement content diminished the *f_tu_* value by up to 22.6% [71]; it was stated that the added SF produced insufficient binder from the calcium hydroxide and SF reaction, which may not compensate for the replaced cement; thus, a lower C-S-H quantity was formed.

### 4.3. Discussion of the Results

For mixes integrating RFCA and RFNA, the diagonal tensile strength and compressive strength were slightly reduced, due to the pores in the ITZ. Conversely, in the mixes integrating basaltic RCP and AMF, the amount of cement was reduced; then, the compressive strength and tensile strength were initially reduced. The pozzolanic effect of the recycled materials may have enhanced the bond between the concrete particles and increased their tensile strength, while the voids existing in the recycled materials had a greater effect on decreasing the concrete’s strength. When the basaltic RCP and AMF partially replaced the cement, the amount of cement was reduced and the amount of C-S-H in the ITZ was reduced. Afterward, the C-S-H amount increased with time, and the ITZ densified with time. As the cement hydration was faster than that of the basaltic powders [78,79], the impact of the basaltic powders on the results was insignificant. At 56 days, the basaltic powders existing in the RFCA, RFNA, RCP, and AMF samples enhanced both the tensile and compressive strength, can offer nearly the same compressive strength as the control mixes, and sometimes enhanced the diagonal tensile strength. The basaltic powders densified the ITZ as it acted as a filler and increased the C-S-H amount. The effect of basaltic powders on the concrete strength and the ITZ was also reported in [62].

### 4.4. Correlation between Tensile and Compressive Strengths

**Figure 13:**
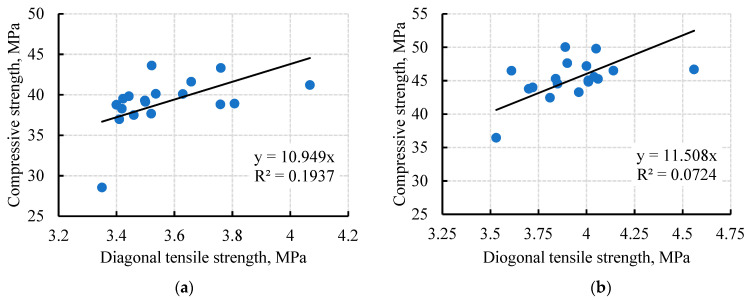
Correlation between the tensile and compressive strengths: (**a**) At 28 days; (**b**) At 56 days.

The relationship between the diagonal tensile strength and compressive strength of the tested cubes at 28 and 56 days is shown in Figure 13a,b, respectively. At 28 days, the relationship between the tensile and compressive strengths was linear (y = 10.949x, with a correlation R^2^ = 0.995). Consequently, at 56 days, the relationship between the tensile and compressive strengths was also linear (y = 11.508x, with a correlation R^2^ = 0.996), where y, x, and R are the compressive strength, the diagonal tensile strength, and the correlation between the tensile and compressive strengths, respectively. From the previous relationships, it was obvious that the diagonal cubic strength was linearly raised as the compressive strength was enhanced with the high correlation, depending on the replacement recycled materials.

### 4.5. Water Absorption

**Figure 14:**
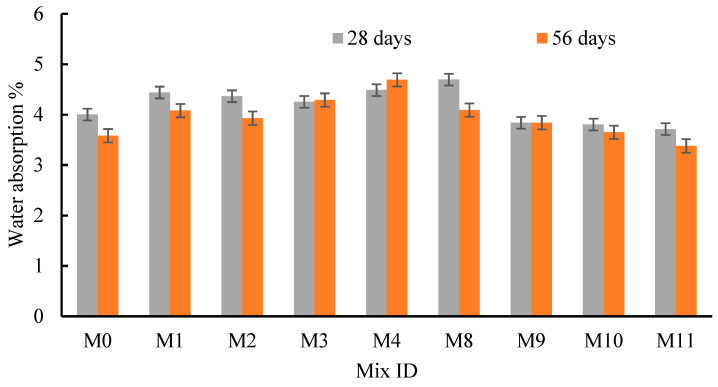
Effect of RFCA and RFNA on the water absorption of concrete.

**Figure 15:**
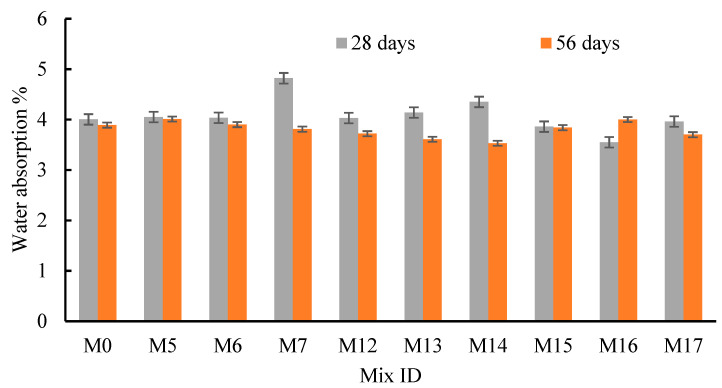
Effect of RCP, AMF, and SF on the water absorption of concrete.

To study the effects of RFCA, RFNA, RCP, AMF, and SF on the concrete durability, the WA for all mixes was found at 28 and 56 days (Figure 14 and Figure 15). All mixes generally experienced a lower WA after 56 days of curing than their WA after 28 days of curing except in the case of M3, M4, M9, and M16 (Figure 14 and Figure 15). The cement was extremely hydrated; the C-S-H gel increased, and the voids diminished. Moreover, at the same Tc, the WA was dependent on the source, fitness, and percentages of the replacement recycled or mineral materials. Similarly, mixes M5-M8 exhibited higher WA values than mix M0 at 28 and 56 days (Figure 14). The two mixes, M2 (RFCA = 40%) and M3 (RFCA = 60%), experienced the lowest WA at 56 and 28 days, respectively. Conversely, as the RFNA replaced sand, M11 (RFNA = 80%) had the lowest WA, either at 28 days or at 56 days. Moreover, the WA of the RFNA mixes decreased as the RFNA percentage increased at 28 and 56 days (Figure 14). Except for M12, the WA for RFNA mixes yielded lower values than that of M0 at 28 days. Consequently, at Tc = 56 days, only M11 experienced a lower WA value than that of M0 (Figure 14). The WA for RFCA or RFNA mixes may depend on the powder percentages and their pozzolanic effects. Conversely, as RCP replaced cement by 5% and 10%, the WA at 28 and 56 days was slightly affected, while as the RCP increased to 20%, the WA was increased (Figure 15). Up to 10% RCP, RCP could restore the same C-S-H gel as cement, while at 20% RCP, the cement hydration may be affected, and the C-S-H gel reduced. Consequently, the WA for AMF slightly increased as the AMF percentage increased (Figure 15); the highest WA values were at 20% AMF. Moreover, the WA of SF mixes was slightly decreased at 28 days, when the SF percentage was less than 20%, while at 56 days, the WA of M15 and M16 was slightly higher than that of M0 (Figure 15). Comparing the effect of RCP, AMF, and SF on the WA demonstrated that SF showed slightly decreased WA values, compared to the RCP and AMF. This may be because of the higher pozzolanic effects and surface area of SF, compared to those for the RCP and AMF. In addition, as the Tc increased from 28 to 56 days, the WA decreased for all mixes integrating RCP, AMF, and SF, except for M16 (Figure 15). The low density and cracks in the RFCA increased the WA of RAC [80].

### 4.6. SEM and EDS Analysis

**Figure 16:**
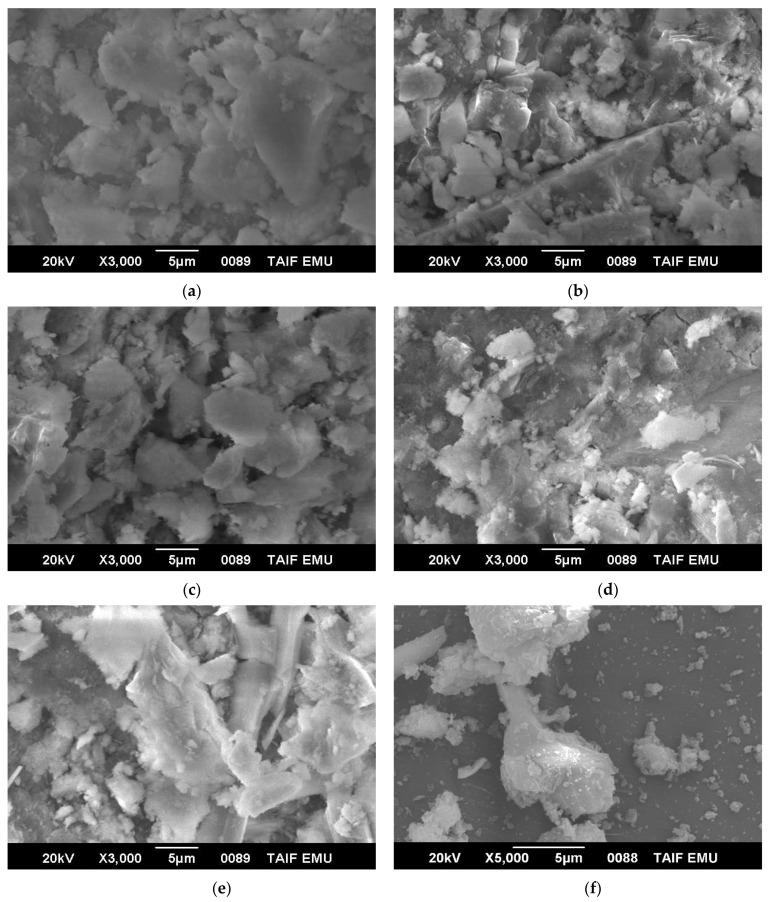
SEM of all tested concrete mixes: (**a**) M2 (40% RFCA); (**b**) M9 (40% RFNA); (**c**) M6 (10% RCP); (**d**) M13 (10% AMF); (**e**) M16 (10% SF); (**f**) M0.

The SEM images of mixes incorporating 40% RFCA, 40% RFNA, 10% RCP, 10% AMF, and 10% SF were evaluated (Figure 16). The figures showed the availability of the C–S–H gels in all the mixes. The basalt particles were covered with some of the hydrated products. For RFCA mixes, the pozzolanic reaction of basalt was greater, due to the basalt and mortar powder attached to the basalt particles; therefore, the hydrated products increased (Figure 16). The rated products decreased for RFNA mixes compared to RFCA mixes as they contain lower quantities of attached basalt powders than RFCA mixes (Figure 16a,b). Consequently, for the same reason, the RCP mixes had higher hydrated products compared to RNA mixes (Figure 16c,d). The unreacted basalt particles may act as fillers. Figure 16e shows the SEM image of the SF mix, while Figure 16f shows the SEM image of the control mixes. Particles of fine aggregates were covered with hydrated products while others were unreacted. Moreover, the basalt particles in the control mixes were partially coated with thick hydrated products (Figure 16). The SEM analysis showed that the C-S-H did not completely cover the basalt particle surface in all mixes. The mixes integrating RFCA had the densest ITZ (Figure 16).

**Figure 17:**
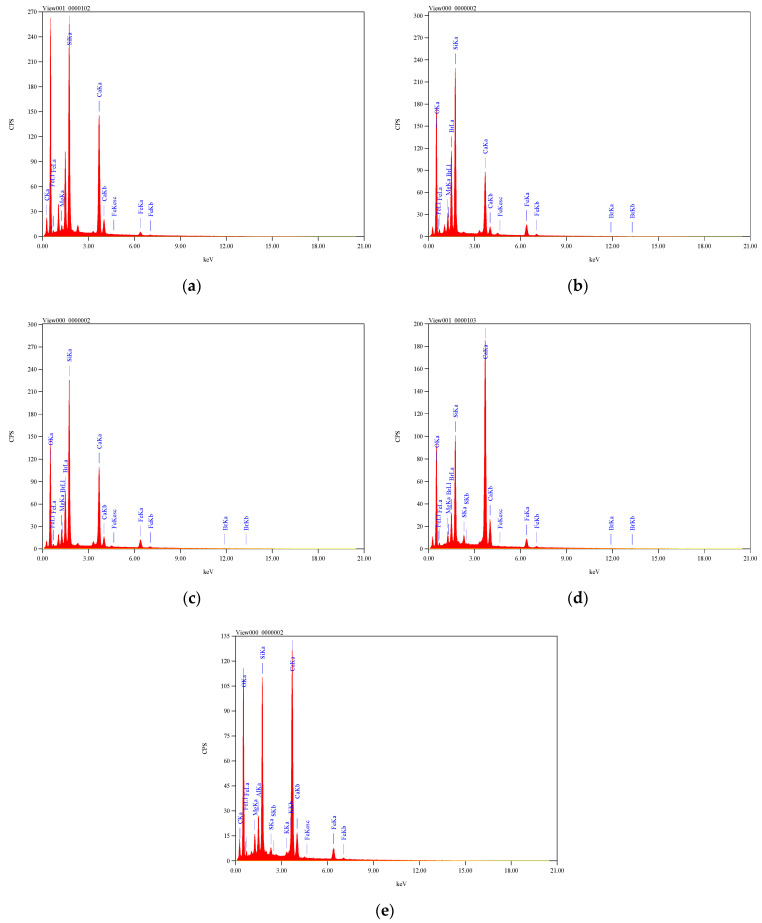
EDS results of the tested mixes: (**a**) M2 (40% RFCA); (**b**) M9 (40% RFNA); (**c**) M6 (10% RCP); (**d**) M13 (10% AMF); (**e**) M16 (10% SF).

The EDS analyses of the RFCA, RFNA, RCP, AMF, and SF mixes are shown in Figure 17. The ions may be exchanged locally between the minerals on both the C-S-H phase and basalt particles. The calcium ions were diffused from the pore solution and replaced the magnesium. Moreover, the magnesium ions diffused into the C-S-H gel. This ensured that the chemical interactions happened at the basalt particle’s surface [59,62,81]. Conversely, the growth of silicate ions in the ITZ helped to form additional C-S-H gel. This extra C-S-H gel could enhance the cement matrix of the mix. Besides this, the ITZ strength increased as the outer aggregate layer was modified by the chemical reactions [81,82,83,84,85,86,87]. Comparing the magnesium, calcium, and silicate contents in the tested mixes showed that the 40% RFCA mixes diffused the highest contents of both calcium and silicate (Figure 17). The high calcium and silicate contents enhanced the ITZ and enhanced the concrete properties compared to the other tested mixes. The high content of calcium and silicate contents in the RCP, AMF, and SF mixes could almost retain the concrete strength of the mixes with lower cement contents, compared to the control mix.

## 5. Conclusions

- Increasing the curing time (Tc) augmented the compressive and tensile strengths regardless of the concrete components. The concrete strength increased at a high rate until 28 days had passed, then the rate decreased afterward because the cement was highly hydrated during the first 28 days.
- The source, type, and content of the recycled fine basaltic aggregate and powders had a great effect on the concrete’s properties. Consequently, as RFCA replaced sand by 40%, the RFCA achieved about 99.5% of the M0 compressive strength at 56 days as RFCA may integrate more basaltic and cement powders than RFNA.
- As RCP, AMF, and SF were partially replaced with cement, the compressive strength was reduced by dissimilar percentages, depending on the additional amount of C-S-H gel produced in the mix. At 28 days, for the 10% RCP mix, the compressive strength was about 99.3% that of the M0 sample strength.
- The tensile strength greatly depended on the source, type, and percentage of the fine recycled aggregates and powders, apart from the curing ages. As the RFCA and RFNA replaced the sand, the tensile strength was augmented. Conversely, as the RCP, AMF, and SF replaced the cement, the tensile strength was enhanced.
- The WA diminished with the increased curing time or testing ages. The WA of the AMF mixes decreased as the RFNA percentage increased, while the RFCA mixes showed the opposite trend. Moreover, compared to M0, the mixes integrating RCP, AMF, and SF generally showed small WA differences. Furthermore, mixes integrating 10% and 20% SF experienced the lowest values of WA.
- The SEM analysis showed that the C-S-H did not completely cover the basalt particle surface for all mixes. The mixes integrating RFCA had the densest nature, in terms of the ITZ.
- The RCP and AMF had the same potential pozzolanic reactivity as SF, but they retarded the hydration reaction. The basaltic RCP and AMF contents could consume Ca(OH)_2_ and produce additional C-S-H gel.
- Comparing the magnesium, calcium, and silicate contents in the tested mixes showed that the 40% RFCA mixes diffused the highest contents of both calcium and silicate. The high calcium and silicate contents enhanced the ITZ and enhanced the concrete properties, compared to the other tested mixes.
- As the concrete properties obtained from both recycled and natural aggregates are nearly the same, the use of recycled aggregate to partially replace the natural ones reduces the extraction energy needed. Conversely, using concrete micro fines to partially replace OPC lessens the CO_2_ emissions that result from OPC production. Therefore, using recycled materials to replace both natural aggregate and cement is an effective way to lessen construction costs and their environmental impact.

## Figures and Tables

**Table 1:** The physical properties of natural and recycled aggregate.

Physical Properties	Crushed Basalt	Sand	RFCA	RFNA
Apparent specific gravity (kg/m^3^)	2.85	2.63	2.64	2.83
Bulk specific gravity (SSD) (gr/cm^3^)	2.76	2.28	2.15	2.78
Bulk specific gravity (GD), (gr/cm^3^)	2.71	2.03	1.86	2.74
Water absorption (%)	1.77	10.96	13.82	13.83
Moisture content (%)	0.93	2.73	2.37	2.22

**Table 2:** The chemical compositions of OPC, RCP, AMF, and SF.

Item	OPC *	RCP	AMF	SF *
SiO_2_	21.28	66.23	60.42	90.2
CaO	64.64	-	4.59	-
AL_2_O_3_	5.60	9.61	15.57	1.2
Fe_2_O_3_	3.42	11.84	14.60	1.8
MgO	2.06	12.32	4.83	6.2
SO_3_	2.12	-	-	-
L.O.I	0.88	-	-	0.6

* From supplier data.

**Table 3:** Concrete mixture proportions per 1 m^3^.

Group	Mix ID	RCA (%)	SF (%)	RCP (%)	AMF (%)	Aggregates (kg)			Water (kg)	Add. Water (kg)	Cement (kg)
						Sand	Basalt	RFCA or RFNA			
Control	M0	0	0	0	0	692	1094	0	215	0	430
	Recycled concrete wastes										
RFCA	M1	20	0	0	0	553.6	1094	138.0	215	4.1	430
	M2	40	0	0	0	415.2	1094	275.8	215	8.3	430
	M3	60	0	0	0	276.8	1094	413.6	215	12.4	430
	M4	80	0	0	0	138.4	1094	551.5	215	16.6	430
RCP	M5	0	0	5	0	692	1094	0	215	0	409
	M6	0	0	10	0	692	1094	0	215	0	387
	M7	0	0	20	0	692	1094	0	215	0	344
	Recycled natural basalt wastes										
RFNA	M8	20	0	0	0	553.6	1094	128.6	215	4.3	430
	M9	40	0	0	0	415.2	1094	257.2	215	8.7	430
	M10	60	0	0	0	276.8	1094	385.8	215	13.0	430
	M11	80	0	0	0	138.4	1094	514.4	215	17.4	430
AMF	M12	0	0	0	5	692	1094	0	215	0	409
	M13	0	0	0	10	692	1094	0	215	0	387
	M14	0	0	0	20	692	1094	0	215	0	344
	Industrial wastes										
SF	M15	0	5	0	0.5	692	1094	0	215	0	409
	M16	0	10	0	0.5	692	1094	0	215	0	387
	M17	0	20	0	0.5	692	1094	0	215	0	344

**Table 4:** The concrete compressive strengths for all mixes at 7, 28, and 56 days.

Group	Mix ID	Specimen No.	*f_cu,_*_7_ MPa	Mean (*σ_µ_*) MPa	*µ*_7_ %	*f_cu,_*_28_ MPa	Mean (*σ_µ_*) MPa	*µ*_28_ %	*f_cu_*_,56_ MPa	Mean (*σ_µ_*) MPa	*µ*_56_ %	*µ*_28/7_ %	*µ*_56/28_ %
Control	M0	1	35.6	34.1 (±1.34)	0.0	43.5	43.6 (±0.10)	0.0	52.5	50.0 (±2.72)	0.0	28.0	14.7
		2	33.1			43.7			50.5				
		3	33.5			43.6			47.1				
Recycled concrete wastes													
RFCA	M1	1	30.6	29.8 (±0.80)	12.5	40.54	39.8 (±1.22)	8.7	42.8	44.5 (±0.00)	11.0	33.6	11.8
		2	29.0			40.50			45.5				
		3	29.8			38.40			45.2				
	M2	1	29.8	29.0 (±0.80)	14.8	39.63	39.5 (±0.21)	9.4	47.9	49.8 (±0.00)	0.50	36.1	26.0
		2	28.2			39.61			54.7				
		3	29.0			39.26			46.5				
	M3	1	32.5	33.7 (±1.20)	1.0	40.64	41.6 (±2.46)	4.6	46.5	45.3 (±1.28)	9.50	23.3	8.8
		2	34.9			44.40			43.9				
		3	33.8			39.77			45.3				
	M4	1	32.4	32.3 (±0.10)	5.2	39.98	40.1 (±0.57)	8.1	45.7	45.0 (±1.32)	10.0	24.1	12.4
		2	32.3			40.70			43.5				
		3	32.2			39.58			45.9				
	M5	1	32.02	32.0 (±0.06)	6.0	38.73	38.8 (±0.50)	11.0	44.6	44.8 (±0.55)	10.4	21.2	8.7
		2	31.95			39.33			45.4				
		3	32.07			38.34			44.4				
RCP	M6	1	22.34	22.9 (±0.84)	32.7	43.39	43.3 (±0.32)	0.70	46.9	47.6 (±0.67)	4.8	88.8	11.1
		2	23.90			43.57			48.2				
		3	22.56			42.94			47.7				
	M7	1	22.89	21.6 (±1.24)	36.7	39.70	38.3 (±2.32)	12.2	41.2	42.4 (±1.07)	15.2	77.4	11.4
		2	20.42			39.52			43.0				
		3	21.41			35.59			43.0				
Natural aggregate wastes													
RFNA	M8	1	29.0	28.8 (±4.79)	15.5	39.4 40.8 40.1	40.1 (±0.73)	8.0	45.7	45.5 (±0.67)	9.0	39.3	14.3
		2	28.6						44.8				
		3	20.5						46.1				
	M9	1	21.5	21.4 (±0.60)	37.1	39.5	39.3 (±1.54)	9.9	43.3	43.3 (±0.34)	13.5	83.3	13.2
		2	20.8			37.7			43.6				
		3	22.0			40.7			42.9				
	M10	1	32.9	29.6 (±3.46)	13.1	40.8	41.2 (±0.38)	5.5	47.3	46.5 (±1.12)	7.1	39.2	11.9
		2	31.2			41.6			45.2				
		3	26.3			41.2			46.9				
	M11	1	26.0	26.8 (±0.78)	21.3	39.3	38.9 (±0.12)	10.8	45.1	46.7 (±1.47)	6.7	45.2	19.8
		2	26.9			39.3			48.0				
		3	27.5			39.1			46.9				
AMF	M16	1	35.36	31.82 (±4.88)	6.6	39.46	39.11 (±0.55)	10.3	42.87	43.98 (±0.97)	12.1	22.91	11.2
		2	25.60			39.40			44.71				
		3	30.50			38.48			44.35				
	M17	1	26.70	26.50 (±1.21)	22.2	38.85	38.75 (±0.52)	11.5	48.70	46.47 (±2.25)	7.1	46.23	12.2
		2	27.60			38.23			44.21				
		3	25.20			38.64			46.50				
	M18	1	15.93	17.45 (±2.13)	58.5	32.89	28.54 (±0.94)	34.3	36.35	36.45 (±0.51)	27.1	63.55	12.8
		2	16.54			31.11			36.00				
		3	19.88			31.48			37.00				
Industrial wastes													
SF	M19	1	27.78	27.67 (±0.32)	18.8	37.70	37.66 (±0.14)	13.6	45.00	45.27 (±1.42)	9.5	73.5	20.2
		2	27.31			37.50			44.00				
		3	27.92			37.78			46.80				
	M20	1	26.58	26.54 (±0.28)	22.1	36.81	36.96 (±1.86)	15.2	47.70	47.17 (±0.50)	5.7	71.8	27.6
		2	26.24			38.89			46.70				
		3	26.80			35.19			47.10				
	M21	1	18.60	18.20 (±0.53)	46.6	35.79	37.48 (±0.1.81)		42.00	43.77 (±1.59)	12.5	48.6	16.8
		2	17.60			37.26		14.0	44.20				
		3	18.40			39.39			45.10				

$\mu_{Tc}\%=\frac{f_{cu,Tc}\left(any\;mix\right)}{f_{cu,Tc}\left(Mix0\right)}\times 100$ where *T_c_* = 7, 28, and 56 days.

**Table 5:** The concrete tensile strength for all mixes at 28 and 56 ages.

Group	Mix ID	Specimen No.	*f*_*tu*,28_ MPa	Mean (*σµ*) MPa	*µ*_*t*28_ %	*f*_*tu*,56_ MPa	Mean (*σµ*) MPa	*µ*_*t*56_ %	*µ*_*t*56/28_ %	*f*_*tu*,28_/*f*_*cu*,28_ %	*f*_*tu*,56_/*f*_*cu*,56_ %
Control	M0	1	3.48	3.52 (±0.05)	0.00	3.92	3.89 (±0.03)	0.00	10.45	8.08	7.78
		2	3.58			3.88					
		3	3.51			3.87					
Recycled concrete wastes											
FRCA	M5	1	3.66	3.44 (±0.20)	−2.24	4.00	3.85 (±0.13)	0.26	13.27	8.65	8.76
		2	3.29			3.78					
		3	3.38			3.78					
	M6	1	3.27	3.42 (±0.20)	−2.88	3.87	4.05 (±0.18)	5.40	19.78	8.67	8.24
		2	3.34			4.24					
		3	3.65			4.05					
	M7	1	3.67	3.66 (±0.04)	3.87	4.25	4.06 (±0.17)	5.40	12.08	8.79	9.06
		2	3.61			3.96					
		3	3.70			3.96					
	M8	1	3.21	3.63 (±0.36)	3.05	4.20	4.01 (±0.17)	3.09	10.49	9.05	8.90
		2	3.95			3.86					
		3	3.73			3.96					
RCP	M9	1	3.88	3.76 (±0.11)	6.81	4.02	4.01 (±0.03)	3.09	6.68	9.69	8.95
		2	3.66			3.99					
		3	3.74			4.03					
	M10	1	3.90	3.76 (±0.16)	6.81	3.88	3.90 (±0.07)	0.26	3.72	8.68	8.19
		2	3.58			3.97					
		3	3.79			3.83					
	M11	1	3.24	3.42 (±0.16)	−2.92	3.87	3.81 (±0.09)	−2.05	11.44	8.93	8.98
		2	3.47			3.85					
		3	3.54			3.70					
Natural aggregate wastes											
FRNA	M12	1	3.54	3.54 (±0.04)	0.40	4.13	4.04 (±0.08)	3.86	14.3	8.82	8.87
		2	3.57			3.97					
		3	3.50			4.03					
	M13	1	3.88	3.50 (±0.31)	−0.68	4.05	3.96 (±0.08)	1.80	13.2	8.90	9.15
		2	3.25			3.93					
		3	3.56			3.90					
	M14	1	4.28	4.07 (±0.19)	15.50	4.07	4.14 (±0.11)	6.43	1.80	9.88	8.91
		2	4.00			4.26					
		3	3.92			4.08					
	M15	1	3.77	3.81 (±0.10)	8.11	4.62	4.56 (±0.07)	17.23	19.8	9.79	9.77
		2	3.73			4.47					
		3	3.92			4.58					
RNP	M16	1	3.50	3.50 (±0.04)	−0.56	3.61	3.72 (±0.36)	−4.37	6.30	8.95	8.46
		2	3.46			3.43					
		3	3.53			4.11					
	M17	1	3.35	3.40 (±0.04)	−3.50	3.48	3.61 (±0.12)	−7.19	6.20	8.77	7.77
		2	3.40			3.72					
		3	3.44			3.62					
	M18	1	3.98	3.35 (±0.59)	−4.80	3.34	3.53 (±0.19)	−9.25	5.40	11.74	9.68
		2	2.81			3.71					
		3	3.27			3.53					
Industrial wastes											
SF	M19	1	3.52	3.52 (±0.01)		3.86	3.84 (±0.03)				
		2	3.53		0.00	3.81		−1.3	8.90	9.35	8.48
		3	3.53			3.85					
	M20	1	3.31	3.41 (±0.09)		4.00	4.00 (±0.03)				
		2	3.49		−3.30	3.98		2.9	17.5	9.23	8.48
		3	3.42			4.03					
	M21	1	3.35			3.61	3.70 (±0.32)				
		2	3.59	3.46 (±0.12)	−1.90	3.43		−5.0	7.00	9.23	8.45
		3	3.43			4.05					

$\mu_{t,Tc}\%=\frac{f_{tu,age}\left(any\;mix\right)}{f_{tu,age}\left(Mix0\right)}\times 100.$

## Data Availability

Not included; the data presented in this article are obtained from an experimental study conducted by the authors.
